# Solo Fathers and Mothers: An Exploration of Well-Being, Social Support and Social Approval

**DOI:** 10.3390/ijerph19159236

**Published:** 2022-07-28

**Authors:** Catherine Jones, Sophie Zadeh, Vasanti Jadva, Susan Golombok

**Affiliations:** 1 Centre for Family Research, University of Cambridge, Cambridge CB2 3RQ, UK; seg42@cam.ac.uk; 2 Thomas Coram Research Unit, Social Research Institute, UCL Institute of Education, London WC1H 0AA, UK; s.zadeh@ucl.ac.uk; 3 Institute for Women’s Health, UCL, London WC1E 6HU, UK; v.jadva@ucl.ac.uk

**Keywords:** single fathers, surrogacy, well-being, stigma

## Abstract

Research has begun to explore the experiences of single mothers by choice who choose to start a family alone and do so using donated gametes. However, very little is known about the experiences of single fathers by choice, and even less is known about how their experiences might compare to mothers in the same position. This exploratory study of single mothers (*n* = 19) and fathers (*n* = 17) by choice examined mental health and social support among mothers who used sperm donation, and fathers who used egg donation and surrogacy, to become parents. Data relating to their reasoning for pursuing solo parenthood, mental health, and social support were analyzed quantitatively. To further explore fathers’ experiences of being a solo parent, a thematic analysis was conducted exploring their descriptions of social responses to their family type. Regarding parental mental health, no statistically significant differences were found between mothers and fathers, and both groups of parents had sought out supportive networks, both before becoming a parent, and as a single parent. Fathers’ responses indicated that they received both supportive and negative reactions, although they generally perceived the majority of interactions to be positive. However, frequent references made by members of the public, or by the media, to their family type being new or different served to reproduce social scripts about normative family types. The study findings, despite the small sample size, contribute to a new understanding of the well-being and experiences of both mothers and fathers who choose to start their family and parent alone.

## 1. Introduction

In the latter half of the 20th century and the beginning of the 21st century, the number of single-parent families has steadily increased. Despite a levelling off of this increase in recent years, the total number of single parent families remains high; worldwide, the US has the highest rate of single parent families at around a quarter of all families with children under age 18 [1]. In the UK, there are 3 million single parent families [2], representing 15% of families. The majority of single parent households are headed by mothers, although there has been an increase in the number of single father families over time. The proportion of single father households in the UK has remained at around 10% of all single parent families over the last decade [2]. Single parent families usually result from divorce or separation, or they are formed by unpartnered single mothers. However, in recent years, particularly due to legal changes in access to reproductive technologies worldwide, a small but increasing number of single people have actively decided to start a family and parent alone from the outset.

Single mother families have long faced criticism, not least because of a long-lasting assumption about the perceived ‘need’ of children for a father [3]. Further, concerns are often raised about children’s adjustment, and parents’ ability to cope with single parenthood, particularly after parental separation or divorce [3]. Research over the past few decades has indicated that the drop in well-being amongst both mothers and children found in some studies is often attributable to factors associated with divorce rather than single parenthood per se, and in particular, the financial difficulties that may follow divorce or separation [4]. Difficulties among children in single parent families have additionally been found to be related to parental mental health, rather than single parenthood in itself [5]. It is also important to note the significant impact of parental conflict, especially sustained conflict, on child adjustment [6,7]. Longitudinal studies have shown that raised difficulties among children following parental relationship breakdown often lessen over time [8]. Nevertheless, it has also been shown that single parent families tend to receive less social support than two-parent families [9]. Importantly, the challenging circumstances sometimes experienced following divorce are not experienced by all single parent families. The route to single parenthood may play a role in the level of social support received, in experiences of financial strain, and in parent and child adjustment.

Single mothers by choice, who decide to embark on parenthood alone and often do so through assisted reproduction [10], first became the subject of research a few decades ago. Single mothers and fathers by choice are sometimes termed solo mothers and solo fathers; for the purposes of the present investigation, these terms will be used throughout. Murray and Golombok [11] conducted the first study directly comparing 27 heterosexual solo mothers, who conceived using donor sperm, with 50 heterosexual partnered mothers, who also conceived using donor sperm. There were no differences between the two groups of mothers in parental well-being and most of the variables assessing parenting quality, including warmth, yet the single mothers demonstrated slightly lower levels of interaction and sensitivity with their very young children. However, these differences were small, with the authors suggesting that the presence of a partner may have supported the coupled mothers in spending more quality time with their child at this early stage. At the follow-up two years later, the findings presented a different picture. The single mothers experienced greater joy and demonstrated less anger toward their toddler, and their children showed lower levels of emotional and behavioural difficulties compared to children with partnered mothers [12]. It is possible that, after choosing to use donor insemination and become a single parent, the solo mothers felt more positively at this stage compared to the partnered mothers, who used donor insemination after a period of struggling to conceive, and who were less likely to be open about their route to parenthood with others. More recent research, including a multi-method, multi-informant, longitudinal study of solo mothers compared to coupled mothers in the UK, all of whom had used donor conception to conceive their child, produced the same findings—that mothers and children were faring well both when children were in infancy and in middle childhood [13,14]. Both phases of the study indicated that family functioning, such as the level of parenting stress, was more important in predicting child adjustment than the number of parents in the family. Notably, single mothers who choose to parent alone generally report low levels of financial difficulties [15], in contrast to mothers who are single due to divorce. Studies of solo mothers document that the mothers carefully consider their network of friends and family before embarking on the journey alone, and often have close ties with their parents and other family members, who may assist with childcare [16]. Although less research has explored LGBTQ+ solo mothers, one longitudinal study found equivalent parenting quality and child adjustment when comparing lesbian solo mothers to heterosexual solo mothers [17]. Despite the positive outcomes found in research exploring solo mothers and their children, research has also highlighted the problematic portrayals of solo mothers in the media, which firstly serve to perpetuate negative stereotypes, and secondly do not represent the findings of empirical research on this family type [18].

Only very recently has psychological research begun to explore solo father families. The small body of literature so far, which has explored cisgender gay and heterosexual men pursuing fatherhood as a single parent, suggests that these fathers tend to be highly educated and have high incomes [19,20]. Like solo mothers, solo fathers begin their families either through adoption, or through assisted reproduction. Access to either of these routes to parenthood for men varies greatly between different countries. For example, in much of Europe, men cannot access surrogacy. Although non-commercial surrogacy among men is permitted in the UK, parental orders for single men—which transfer legal parenthood from the surrogate to the intended parent—became legal only in 2019 [21]. Surrogacy is more easily accessible through clinics and agencies in the US, although having a child in a country other than their country of residence often presents fathers with many practical and legal challenges. Single fathers often undertake extensive paperwork to gain legal recognition as their child’s father, which is emotionally taxing and time intensive [20].

Social discourse on parenting roles and psychological theories have contributed to assumptions about fathers’ parenting abilities, often positioning fathers as breadwinners and as ‘secondary’ in parenting to mothers. Bowlby’s attachment theory, and his initial writings on the role of maternal love and mothers as primary caregivers [22], set the scene for a focus on mothering and mother-child relationships in psychological research [23,24]. However, in recent decades, greater attention has been paid to fathers. On the whole, findings on parenting quality and parental influences on child adjustment indicate many more similarities than differences between mothers and fathers, and that fathers have the same capacities for high-quality parenting as do mothers [23,25,26,27,28]. Regardless, fathers, particularly those in the primary caregiving role, also face greater stigma and lower social support than mothers [29,30,31].

Two key factors that are considered to be important for positive parent and child adjustment are parent mental health (e.g., [32]), and social support (e.g., [33,34]). These factors are part of the multi-faceted concept of psychological well-being that not only constitutes positive mental health [35], but also encompasses, for example, support and positive relationships with others [36]. In terms of the psychological well-being of parents in different family forms, it remains the case that primary caregiver fathers, and single fathers in particular, face stigma and negative social attitudes, which may have an adverse effect on parental well-being, as well as knock-on effects on wider family functioning. In particular, research on minority stress has demonstrated that the stigma and discrimination faced by LGBTQ+ individuals can lead to greater levels of stress, which, consequently, is associated with health difficulties—both physical and psychological [37,38]. Moreover, despite increased legal recognition of LGBTQ+ parents alongside more accepting social attitudes, gay fathers through adoption or surrogacy have reported experiences of stigmatization in recent research [39]. A study of solo fathers specifically found that increased stigmatization was related to lower sensitivity towards their children by gay and heterosexual single fathers [40]. However, the study did not compare fathers’ experiences and well-being to that of mothers, and whether or not solo fathers face more challenges than mothers in the same position is therefore not yet known. Considering this, it is important to pay attention to the range of influencing factors, including, but not limited to, gender, sexual orientation, age, financial circumstances, and country of residence, that are likely to shape solo fathers’ experiences. To do so, adopting an intersectional lens is pertinent [41].

Very few studies have explored the adjustment of single fathers and their children in elective single father families. In a study by Carone et al. [19], conducted in Italy, which compared the outcomes of single gay fathers through surrogacy, single heterosexual fathers through surrogacy, same-sex male couples through surrogacy, and heterosexual fathers in couples who had used IVF, higher levels of parenting stress were found among both groups of single father families compared to partnered fathers [19]. However, no differences were found regarding depression or anxiety, parenting quality, parent-child relationship quality, or child adjustment. Overall, the families showed positive adjustment, regardless of the number of parents in the family, or the sexual orientation of the parent(s).

More generally, the findings of research on same-sex male couples who used surrogacy to begin their family point to positive parent and child outcomes. A study conducted in the US that compared 40 same-sex male couples through surrogacy to 55 same-sex female couples who conceived their children using donor sperm, found that the children aged 3–9 years old were well-adjusted across both family types and that children in two-father families experienced lower levels of internalizing behaviours than the children in two-mother families [42]. In a study conducted across different sites in the UK, the Netherlands and France, no differences were found in parental well-being between same-sex male couples who used surrogacy, same-sex female couples who used sperm donation, and heterosexual couples who used IVF to conceive their child [43]. Likewise, a study in Israel of same-sex male couples who used surrogacy to start their family found no differences in their psychological well-being compared to heterosexual fathers or to gay fathers who had a child in a heterosexual relationship, yet fathers in same-sex couples through surrogacy expressed greater satisfaction with life and with parenting [44]. These studies show that, across different contexts, fathers in couples who use surrogacy to start a family and their children are faring well. Nevertheless, little is known about the psychological well-being of single fathers who use surrogacy as a route to parenthood. 

The present study, to our knowledge, is the first comparative investigation of solo fathers and solo mothers. As both family types are headed by a single parent, the study enabled the role of parental gender on parental well-being in solo parent families to be examined, controlling for the use of assisted reproduction to start a family. The study also explored differences in reasons for becoming a solo parent and the support available to solo mothers and fathers, both while they were making the decision to become a single parent, and as a single parent. It was hypothesized that solo fathers would experience lower social support than solo mothers due to less accepting social attitudes toward men pursuing parenthood alone [45]. In addition, the study examined single fathers’ experiences of social reactions to their family type, to better understand the experiences of single men who elect to parent alone, given the current dearth of psychological research on solo fathers. Thus, the study set out to explore the following research questions:

1. What are single mothers’ and fathers’ reasons for becoming a solo parent?
2. Does parent gender influence the types of support mothers and fathers seek when deciding to become a solo parent, and the level of support they receive as a parent?
3. How does the psychological well-being of solo fathers compare to solo mothers?
4. What are solo fathers’ experiences of others’ reactions to their family type?

## 2. Materials and Methods

### 2.1. Participants

The sample of solo fathers and solo mothers was drawn from two larger investigations of solo parents by the same research team; from a study of solo mothers in the UK and from an international study of solo fathers. For the purposes of this paper, participants were selected according to the following criteria: (i) the parent had used assisted reproduction to start their family; (ii) the child was in infancy or early childhood; and (iii) the parent had completed questionnaire measures of psychological well-being. Seventeen solo fathers who had formed their family through surrogacy were compared with 19 solo mothers who had started their family through sperm donation. Parents in both family types had become parents alone, although some had begun a relationship following the birth of their child. The single mothers lived in the UK and were recruited through the London Women’s Clinic. All had used their own egg and donor sperm to conceive their child (for full details of the recruitment procedure, see [13]). The mothers were cisgender women and eighteen mothers identified as heterosexual and one mother as bisexual. The fathers lived in Australia, France, Italy, Mexico, Spain, Sweden, Switzerland, the UK and the US, and their surrogacy arrangements had taken place in a range of countries, most commonly in the US. The fathers had all used their own sperm and gestational surrogacy to start their family. The fathers were cisgender men and fifteen fathers identified as gay, one as heterosexual, and one as asexual. They were recruited through Brilliant Beginnings, the Children and Family Court Advisory and Support Service, Circle Surrogacy, Growing Families, Family Equality, and through snowball sampling. 

The original single mother sample [13] comprised a larger group of families with children aged 4–8 years-old. As the single father sample had significantly younger children (with some children still in infancy), only those single mothers with a child aged 4 years-old were included in the present study, in order to match the two types of family for child age. 

#### Demographic information

As shown in Table 1, there was a significant difference between groups in child age, *t*(34) = −2.93, *p* = 0.01. Children of mothers were older (*M* = 50.37 months) than were children of fathers (*M* = 36.12 months). The difference in age between mothers (*M* = 44.05 years) and fathers (*M* = 47.18 years) did not reach significance, *t*(34) = 1.90, *p* = 0.07. There was no difference between family types regarding child gender, the number of children in the family, or the employment status of mothers and fathers. Overall, there were sixteen girls and twenty boys in the sample, single-child families were the most common, and parents were mainly in full-time employment, followed by part-time employment. 

### 2.2. Procedure

Each parent was interviewed and administered questionnaires by researchers trained in the study techniques. At the start of each interview (conducted either in person or online), an information sheet was provided to each parent, opportunities to ask questions were given, and written informed consent was obtained. Following data collection, the interviews were transcribed verbatim with all identifying information removed. The interviews generally lasted around 2 h. Ethical approval for the research had been obtained from the Cambridge Psychology Research Ethics Committee.

### 2.3. Measures

#### 2.3.1. Interview

The interviews covered demographic characteristics of the participants and the participants’ routes to parenthood and experiences as parents (see [13] for further details); the mothers and fathers were asked many of the same questions, although an additional section of the father interview explored fathers’ experiences of starting a family as a single man, reflecting both the similar and unique concerns of each of the larger research projects. For the present paper, only those aspects relating to the research questions were analyzed. Regarding the demographic questions, at the beginning of the interview, mothers and fathers were asked to provide the gender and age of all their household members. An additional questionnaire was provided at the end of the interview which included financial circumstances. The fathers were also asked the open-ended questions of ‘how do you describe your gender identity?’ and ‘how do you describe your sexual orientation?’.

##### Timing and Reasons for Becoming a Single Parent

The first section covered parents’ reasons for becoming a single parent. Participants were asked about the age at which they first wanted to become a parent. This was scored as either ‘had always known’ or the specific age. They were then asked about their main reason for becoming a parent at the time they did. This was coded according to a standardized coding scheme with the following categories: (i) I wanted to be a parent; (ii) Timing felt right; (iii) I was getting older/Felt time was running out; (iv) I was financially secure; (v) All my friends were having children; and (vi) Other.

##### Discussions Leading up to Becoming a Single Parent

During the interview, mothers and fathers were asked whether they had spoken to anyone whilst they were thinking about becoming a solo parent. Their responses were coded as either ‘yes’ or ‘no’ regarding discussions with (i) Family; (ii) Friends; and (iii) Others (including support groups and health professionals).

##### Experiences of Social Reactions to Their Family Type

Fathers were asked two specific questions: ‘How do people usually react when you tell them that you’re a single father?’ followed by ‘Have you experienced any negative reactions from anyone?’. Due to the semi-structured nature of the interview, further follow-up questions and prompts were asked if the interviewer felt more detail was needed to gain a full understanding of participants’ experiences of social responses to their single father status.

#### 2.3.2. Questionnaires

##### The Edinburgh Depression Scale

The Edinburgh Depression Scale (EDS: [46]) was administered to evaluate levels of depression. The scale is comprised of 10 items which are summed to produce a score between 0 and 30. Higher scores reflect high levels of depression. Items include ‘I have felt so unhappy that I have had difficulty sleeping’ and ‘Things have been getting on top of me’. Scores greater than 13 are considered to be above the cut-off for high levels of depressive symptoms [47]. The scale has satisfactory validity and split-half reliability [48].

##### The Trait Anxiety Inventory

The Trait Anxiety Inventory (TAI: [49]) was used to assess anxiety. After relevant items have been reversed scored, the scale, consisting of 20 items, produces scores ranging from 20 to 80, with higher scores indicating greater anxiety. This questionnaire has been shown to discriminate well between clinical and non-clinical groups [49]. Sample items are ‘I lack self-confidence’ and ‘I feel rested’. A score of greater than 40 is considered to indicate high anxiety [50,51]. However, there is no standardized cut-off point for this measure. A meta-analytic review of the TAI reported adequate average reliability coefficients for both test–retest and internal consistency [52].

##### The Parenting Stress Index

The Parenting Stress Index short-form (PSI: [53]) was used to assess the stress parents may feel regarding their parenting role. The 36 items are summed to give a score ranging from 36 to 180, with higher scores reflecting higher levels of parenting stress. Items include ‘My child smiles at me much less than I expected’ and ‘I feel trapped by my responsibilities as a parent’. Total scores of over 90 indicate clinically significant levels of parenting stress. The full-length questionnaire has shown good concurrent and predictive validity, and the short form, used in the present study, is highly correlated with the full-length scale [53].

##### The Multidimensional Scale of Perceived Social Support

The Multidimensional Scale of Perceived Social Support (MSPSS; [54]) was used to evaluate mothers’ and fathers’ perceived social support. The scale is comprised of twelve items assessing the support received from family, friends, and a significant other. The items, scoring from 1 to 7, are averaged, with higher scores representing greater perceived support. Sample items are ‘I get the emotional help and support I need from my family’ and ‘My friends really try to help me’. Scores between 1–2.9 are considered low support, between 3 and 5 as moderate support, and of 5.1 and above are classed as high support [54]. The scale has demonstrated good validity and test–retest reliability [54].

##### Positionality

The interviews and analysis were conducted by two of the authors. The authors are cisgender women who have experience in interviewing LGBTQ+ parents. The authors recognize that, due to their gender identity, they could be considered to have an insider status by the mothers, or outsider status based on other aspects of their identities and experiences. However, the interviewers were likely to have been seen as occupying an outsider status regarding single fathers. The first author, who conducted the analysis, is a non-parent. In line with the principles of reflexive thematic analysis [53], the researchers continually reflected on their positionality and how this may have shaped ideas or assumptions while analyzing the data.

### 2.4. Analytical Approach

A convergent mixed method approach was used. Firstly, frequency counts were made for the age at which parents decided to have a child, reasons for becoming a parent, and the people they discussed their decision to become a single parent with. Comparisons were conducted between the two groups for these variables using Fisher’s Exact tests.

Comparisons between solo mothers and solo fathers on the measures of psychological well-being and social support were conducted. The measures which were normally distributed (social support, depression and parenting stress) were analyzed using independent sample *t*-tests. As anxiety was not normally distributed, a Mann-Whitney U-test was used. Correlations were conducted to explore associations between the mental health and social support variables. Subsequently, in order to compare the solo parents’ well-being to general population norms, Fisher’s Exact tests were carried out to compare the proportion of mothers and fathers scoring above the cut-off points for mental health problems and social support.

Fathers’ accounts of their experiences of how people react when they tell others they are a single father were subject to thematic analysis [55,56,57]. Data on this section were missing for two fathers hence fifteen transcripts were analyzed. After familiarizing with the dataset, line-by-line coding was conducted by the first author. Once all the codes had been collated and condensed, the codes *(n* = 35) were organized into three main themes. Coded extracts were then checked against the themes to assess their suitability and illustrative quotes were selected from across a range of transcripts. The 15-point checklist for establishing quality in reflexive thematic analysis outlined by Braun and Clarke [57] was used in all stages of the process, from transcription, to analysis, and writing up. Further, the lead author, who conducted the thematic analysis, engaged in peer data analysis with other qualitative researchers.

#### Covariates

As the family types differed in the age of the children, correlations were conducted between the age of the child and the outcome variables (presented in Table 2). As child age was not significantly associated with any of the outcome measures, it was not controlled for in the analyses.

## 3. Results

### 3.1. Motivations for Solo Parenthood

Most of the fathers (*n* = 13, 77%) and almost half of the mothers (*n* = 8, 42%) said that they had always known that they had wanted to be a parent, or had thought about it throughout their adult life, rather than remembering a specific age at which they felt a desire to have a child. A Fisher’s Exact test showed that there was a significant difference between mothers and fathers (*p* < 0.05), with fathers more likely to state that they had always known and mothers more likely to state an exact age when they had decided they would like to have a baby. For fathers who had stated a specific age, this ranged from 32 to 42 years (*M* = 36 years), whereas for mothers, it was between 26 and 43 years (*M* = 34 years).

Regarding the main reason for becoming a parent at the time they did, frequency counts and illustrative quotations are presented in Table 3. A Fisher’s Exact test revealed no significant differences in the reasons given by mothers and fathers. The largest portion of parents fell into the category of ‘I wanted to be a mother/father’. These parents attributed pursuing parenthood alone at that point in their life to a strong desire to become a parent. Time passing, the timing feeling right, or a sense of time running out, were cited as the main reason for several mothers and fathers. As the sample is comprised of parents who started their family later than average, it is within this context that the parents described feeling aware of their age, and ready to begin their family, partially due to concerns that they would not be able to be a biological parent otherwise. For fathers, but not for mothers, financial considerations were cited as a key reason; three fathers described that the main reason for having a child at the time they did was because they felt they had built up the economic resources necessary for surrogacy. That the fathers emphasized monetary preparedness more than the mothers makes sense within the context of the high cost of pursuing surrogacy internationally. For one mother, but no fathers, friends having children influenced her decision, illustrating how, for that mother, watching those around her begin families encouraged her to begin a parenting journey. A minority of parents cited other reasons for choosing to become a single parent at the time they did; for example, due to personal circumstances regarding their wider family situation, or so that their older child (who was conceived within a relationship) could have a sibling.

### 3.2. Support from Others

The interviews explored the sources of support the mothers and fathers reached out to in the lead-up to becoming a parent. Table 4 shows the number of mothers and fathers who discussed becoming a single parent with either their family, friends, or others (including support groups). Example quotes from mothers and fathers are also presented.

A Chi-square comparison between the proportion of mothers and fathers who discussed becoming a single parent with their family was not significant, χ^2^(1) = 0.63, *p* = 0.43. Similarly, there was no significant difference regarding discussions with friends, χ^2^(1) = 0.39, *p* = 0.53, or with other people in their lives, χ^2^(1) = 0.42, *p* = 0.53. This finding suggests that mothers and fathers generally had a similar approach to having discussions with those close to them, or with people they felt would be helpful, such as support groups and professionals. Friends were the people participants turned to the most, followed by family, then other sources of support. Only a minority of parents did not reach out to others in the lead-up to their journey to parenthood; for example, when asked if she had discussions with anyone, one mother explained ‘Um, no. Cos it was all so quick and it was something that I knew I couldn’t actually do without, it was one of those things that wasn’t an option… not having a child.’ However, most of the parents reported seeking out advice or initiating discussions with important people in their lives.

As well as examining support whilst considering parenthood, participants’ perceptions of social support once they had become parents were explored using the Multidimensional Scale of Perceived Social Support (See Table 5). An independent samples *t*-test comparing mothers’ and fathers’ scores for perceived social support found no significant difference between them, *t*(34) = 0.55, *p* > 0.05.

A Fisher’s Exact test comparing the number of mothers and fathers classed as having low, moderate or high social support was not significant, *p* > 0.05. The majority of parents were classified as having high levels of support; the percentages of fathers with low, moderate and high social support were 12%, 29% and 59%, respectively, and for mothers, 5%, 32% and 63%, respectively.

Social support was negatively associated with anxiety, *r*(36) = −0.38, *p* = 0.02, such that parents with higher levels of social support were likely to report lower levels of anxiety. However, social support was not significantly associated with depression or parenting stress.

### 3.3. Mental Health

Comparisons were conducted between mothers’ and fathers’ scores for depression, anxiety and parenting stress. The findings are presented in Table 6. T-tests showed no significant differences regarding depression, *t*(34) = −1.56, *p* > 0.05, and parenting stress, *t*(33) = −7.42, *p* > 0.05. A Mann–Whitney U-test found no significant difference between families for anxiety, U = 129, *p* > 0.05. When assessing the proportion of parents above the cut-off for depression, a Fisher’s Exact test was not significant, *p* > 0.05. Two mothers (11%) and no fathers scored above the cut-off. A Fisher’s Exact test examining the number of parents who scored above the cut-off for anxiety was also not significant, *p* > 0.05; five mothers (26%) and two fathers (12%) obtained scores above the cut-off. None of the parents scored above the cut-off for clinically relevant levels of parenting stress.

Depression was significantly associated with anxiety, *r*(36) = 0.71, *p* ≤ 0.001, and parenting stress, *r*(35) = 0.33, *p* = 0.05, and anxiety was also significantly associated with parenting stress, *r*(35) = 0.38, *p* = 0.03. This indicates that parents who reported raised levels on one of the mental health measures were also more likely to experience other mental health difficulties.

### 3.4. Fathers’ Experiences of Reactions and Responses to Their Family Type

A thematic analysis of fathers’ reports of experiences of social reactions to being a single parent produced three main themes: (i) joy; (ii) judgement and (iii) intrigue. The three themes were captured in fathers’ reports across the sample as a whole.

#### 3.4.1. Joy

The first theme of ‘Joy’ encompasses the reactions fathers typically reported experiencing when others first learned that they were single fathers, and particularly when others understood that they had chosen to become a single father. Many of the fathers felt that when they discussed their family type, they generally received a positive response: “The majority is extremely positive and extremely supportive, you know, people calling to support and to help.” It was quite common for fathers to report that the important people in their lives, such as friends and family, were supportive, such as one father recalling that when people found out about him becoming a parent alone they were “Normally excited and pleased for me”, and that the few who reacted negatively were more likely to be people on the periphery of his social circle. Similarly, another father recalled “I never got any [negative responses] from people that are close to me.”. Another father’s comments exemplified this; “it doesn’t necessarily make a difference to most people that I know what sex or what gender or what a parent is, it’s about being there for your child, the people in my circle have that view”. Some fathers experienced encouragement and approval from those who knew the journey they had been through to become a parent: “they all know that I’ve been wanting to be a father for so many years, you know, so they’re so so genuinely super happy for me”.

#### 3.4.2. Judgement

While reports of direct disapproval or criticism were infrequent, such stigmatizing reactions were sometimes mentioned; one father described a challenging and uncomfortable interaction with a friend, “He could not really approve of it, but he was sort of…there was something a bit…strange and a little offensive in what he was saying, there was a lot of judgement.” Another father felt supported as a single father, but faced prejudice when discussing the route to single parenthood he took; “there’s people that they don’t understand surrogacy, they don’t support it. About being a single dad no, probably everybody is having the same [positive] reaction”. A couple of the fathers also described interactions with public services that had been challenging. However, among the types of negative interactions discussed, strangers were more likely to make judgements than were people who knew the fathers well, with the fathers describing incidents founded in gendered assumptions: “the shop assistant saying oh, is it mother’s day off”. When anticipating negative reactions, sometimes fathers turned to strategies to avoid prejudice, such as concealing their true identity; “I just got to a point where I got sick of people asking where his mum was and that I wasn’t capable and stuff, and…I used to say oh, she’s … working”.

Some fathers also mentioned negativity within the media and on social media: “I’ve been reading things on Facebook from friends that are closed and closed-minded.” Another father commented, “the majority, 90% is supportive, but then there’s been quite a lot…a few of bad complaints… …about me being selfish or something like that but the only bad one really is yeah, from the media.”

#### 3.4.3. Intrigue

The third and final theme to emerge from the fathers’ accounts was intrigue; capturing the fact that the fathers felt that many people viewed their family as novel. Solo fathers differ in several ways from the ‘traditional’ image of the family, comprised of two heterosexual, cisgender parents and their biological children [58], and by showing admiration and surprise at this, others’ reactions reaffirmed fathers’ deviation from this image. For example, one father said, “I sense kind of a ‘wow’ effect” upon explaining to others that he chose to be a single father. Similarly, another father reported that people “…think I’m brave,”, and another stated that others’ reactions suggested that, “They think you’re brave and they always think it’s a bit strange that a man’s doing it.” These fathers all experienced similar reactions from others that emphasized the rarity of men parenting alone; one father reported that regarding others’ reactions, that “majority it’s wow, really, can you do it like that? Yeah, like astonished…but again, you know, the aftermath of that sort of astonishment is like…that’s amazing and that’s so perfect for you”. Thus, although not explicitly negative, these reactions were sometimes perceived by fathers as emphasizing their non-normative family circumstances. This was reflected in another father’s account, whereby he recalled that one woman said to him, “Well I’ve never heard of that before, I didn’t even know that was possible, how novel”, and the father reported, “She just kept saying how novel.”

Several fathers explained that because the structure of their family was perceived to be uncommon, people often asked questions: about them being a single father, about their child not having a mother, or about the process of surrogacy itself. Some of the fathers explained that they thought some of the questions asked by others were not rooted in criticism; instead, they read them as attempts to gain a better understanding of their route to parenthood and family set-up; “ [Someone] was asking me lots of questions about how and oh and all of this, but it wasn’t negative, it was just people trying to understand and why on earth would I do that.” Similarly, another father said that in his situation, “It’s a baby, as a guy with a baby, you do probably get asked a lot more ‘where’s the mum, where’s the mum?’ and that.” One father reflected on how it had become easier for him now that his child was a little older; “A little baby, everyone’s coming up to you and from the security people at the airport to whatever, you’ve got this baby and you’re a man and there’s something wrong with that picture, it’s not…there’s got to be a woman somewhere, so…people asked questions, whereas now nobody sees it as any different.” The questions fathers were asked were all based on assumptions about mothers as primary caregivers and resulted in fathers having to explain their familial circumstances to others, for example, one father explained that he tells others “He hasn’t got a mother, he’s only got me as a parent.” Occasionally, the fathers decided to respond to such questions in order to highlight family diversity; “My [family member] has taken an exception to the fact that I do actually…sometimes tell people that I’m a single gay dad and…but for me, that’s actually partly sort of…she sees it that I’m painting a target on my back and inviting trouble, I view it that actually it’s me creating visibility so that people are actually aware that families do come in all shapes and sizes.”

## 4. Discussion

The present study found that mothers and fathers shared similar reasons for becoming a solo parent and sought out similar sources of support to discuss their decision about their route to parenthood. Exploratory quantitative analyses of parental social support, depression, anxiety, and parenting stress also showed comparable results regardless of parent gender, despite the prevailing assumption that solo fathers would face more challenges and feel less accepted than mothers [41]. Given the limited literature on solo fathers, a thematic analysis was also conducted to explore solo fathers’ experiences of social reactions to their parenting status and family type. Qualitative findings showed that the fathers generally felt they received positive reactions from other people, with negative responses mainly being isolated incidents, yet interactions with the general public were also described as characterized by others’ interest in, and intrigue about, their families. Together, these findings contribute to a better understanding of the experiences of fathers who decide to start a family and parent alone; on the whole, fathers were doing well and did not show any differences in terms of levels of well-being and social support to mothers in the same position. Yet, findings also suggest that more needs to be done to ensure that the media and general public are accepting of family diversity, and in particular, of solo parenting by men.

This study is among the first to explore solo fathers’ reasons for starting a family as a single parent, and to compare these reasons to those of mothers in the same position. The reasons given by mothers and fathers for choosing to embark on parenthood alone largely reflect the reasons cited by solo mothers in previous research [15,16], whereby increasing age and a desire to have a child took precedence over a wish to have a child within a relationship. This demonstrates that, although there is a long-lasting assumption that women have a more active desire to become a parent than do men [45], these men also prioritized their desire to become a father and went to great lengths to start their family. Importantly, these findings, as well as the findings of research on same-sex male couple families (e.g., [26]), challenge gendered assumptions about the desire to become a parent.

In keeping with the small body of literature on solo parents [13,14,19], the mothers and fathers in this study reported high levels of psychological adjustment and showed no differences compared to general population norms and cut-off points for the measures of well-being. The positive well-being reported by the parents may relate to their fulfilling a long-term desire to become a parent, as exemplified in the finding that most of the parents had wanted to become a parent for many years. Indeed, the parents in the present study described feeling highly motivated to become a single parent and realizing a desire that was of great importance to them. Such findings arguably relate to wider research on couples who use assisted reproduction to start their family, which indicates that, likely due to being highly motivated to become a parent and going to great lengths to do so, these parents often demonstrate resilience to the challenges posed to their well-being during the transition to becoming a parent [59]. While the sample size was small and, therefore, it is possible that small differences between the groups were not detected, the exploratory analyses suggest that parental gender does not have a significant influence on the mental health of solo parents. Such findings have extended those of previous studies, which have compared fathers’ well-being to that of other fathers [19] or explored fathers’ reports from a qualitative perspective only [60]. In addition, the similarly high levels of social support reported by solo mothers and fathers in the present investigation add further weight to the view that solo parents have different experiences to those of single parents due to unforeseen circumstances [61].

The qualitative findings of this study suggest that single fathers generally experienced social support and social approval. However, in line with other literature on solo parents [62], they were also found to have faced stigmatizing interactions. These were experienced particularly with strangers. The fathers had also been exposed to negative representations of their family type in the media, in addition to being the target of questioning by others. Such findings paint a nuanced picture of how fathers who choose to start a family alone are perceived socially. They highlight that the fathers, despite receiving support and praise, also experience both distal stressors [37,38] in the form of stigmatizing interactions such as being scrutinized by airport security, and proximal stressors, such as anticipating negative reactions hence concealing their identity as a single father who chose to start a family alone. That some of the fathers, and the fathers’ extended family, felt hesitant about being open about their identity as a single gay father demonstrates the importance of considering intersectional stigma when exploring the experiences of solo parents, in order to better understand how different aspects of the fathers’ identities may influence their parenting experience. Such findings are similar to those from other research; gay fathers in different family constellations have reported stigmatizing interactions [39,63]. In particular, the finding in the present study that the solo fathers experienced both positive and negative reactions reflects that of a study of gay fathers in which the fathers reported simultaneously experiencing stigma and inclusion at their child’s school [64]. The present study, and previous research, suggests that although solo fathers show positive adjustment, their experiences are still affected by facing prejudice in their parenting role.

Secondly, the findings from the thematic analysis echo previous research on primary caregiver fathers in studies of stay-at-home fathers, who have been shown to have received extensive praise [65], simultaneously highlighting approval from others, alongside evidencing societal assumptions (i.e., that it is not the norm for fathers to be highly involved parents). Further, the questions the fathers in the present study faced, particularly those relating to where a mother was, suggest that not only is greater social value placed on parenthood within a couple, but that the social acceptability of single parenthood may reflect gendered assumptions. The experiences of the fathers in the current study hence reflect those of a small study of single gay fathers in Israel, in which fathers felt that society placed much greater value on coupled parenthood [60].

One might expect that the findings relating to stigmatizing interactions in the present study would relate to fathers’ well-being. However, given that the exploratory quantitative findings indicated that fathers were doing well, it seems that these experiences were not consequential for psychological health. While further research with a larger sample of fathers—with more varied social experiences—would be required to explore this quantitatively, a possible explanation for the present findings is that the fathers were resilient to minority stressors due to the resources, both individual and community-based, that they had access to in terms of their individual characteristics, economic situation, and social network [66]. Indeed, the majority of parents in this study perceived that they had either moderate or high levels of social support. Social support helps individuals to face challenges and can help buffer against stressors [67], for example, through lessening the impact of parenting stress [34]. In the context of the present study, it is likely that the social support parents received was beneficial both in terms of parenting stressors, and in buffering against experiences of stigma or discrimination as a single parent. It appears that the fathers had carefully chosen their networks to make sure that they had people in their lives who would support and help them: both in their role as a single father, and in feeling accepted. It is important to note, however, that the sample was comprised of fathers who were affluent, particularly as many had accessed high-cost surrogacy transnationally, and that the mothers were also relatively affluent and financially secure. It has previously been suggested that the positive outcomes demonstrated by solo mother families reflect the greater influence of parental financial situation than family structure on family outcomes [68]. The findings of the present study may not be replicated in samples with different socioeconomic circumstances where parents do not have the resources to ensure that they will receive support from others, or to choose the form that this support will take.

That mothers and fathers drew upon advice from family, friends, support networks, and professionals in the lead up to their decision to pursue parenthood alone, and then also reported high social support as parents, reflects previous sociological thinking about solo mothers [69], which conceptualized mothers as actively creating and drawing upon extended networks of kin while raising their children on their own, yet extends previous literature by providing novel information about the support networks sought by solo fathers. The findings of this study further suggest that not only family and friend relationships are important—community sources of support may also be relevant. This was the case for the mothers in the study—based in the UK—and for the fathers—based in a number of different countries. Given that the legal status of solo fathers in such countries may be different, it is noteworthy that levels of interpersonal and professional support were nevertheless generally high. Such findings potentially point to the importance of distinguishing between different forms that stigma [70] towards solo father families may take, in addition to examining the nuances of their social experiences and social responses to their familial circumstances. Future research needs to further explore fathers’ experiences of interacting with public services, such as healthcare and education providers, to assess the quality of structured, and/or state-funded support, they may be receiving. Although the social experiences of the fathers in this study were generally positive, their experiences of feeling judged and as targets of interest and intrigue would suggest that more needs to be done to facilitate single fathers feeling accepted and represented in societal discourse on the family, which would likely have an important effect on the types of interactions single fathers have, and the way they are treated.

Due to the small sample size, the findings from this study should be interpreted with caution. However, as the first to directly compare parental well-being and social support between solo fathers and solo mothers, this study provides new insight into the question of whether gender plays a role in the adjustment of parents who start a family alone. Aside from the small sample size, there are other limitations which are worth reflection. It is important to note that the research, like previous studies, only included cisgender parents. The majority of the fathers identified as gay andthe majority of the mothers identified as heterosexual, so little is known about the experiences of solo parents who are trans, or non-binary, or bisexual. Only one study so far has explored the experiences of trans single parents [71]. In addition, the two samples in the present study were not perfectly matched, not only because of the lower average age of children in the solo father families, but also because the mothers were recruited in the UK and the fathers internationally. The cost of treatment for surrogacy varies drastically between countries, as do legislation and social attitudes toward different types of families. Hence, samples from the same country that directly compare single mothers and fathers who choose to start a family and parent alone should be the focus of future inquiries. Lastly, future research should explore the adjustment of children in solo father families and child perspectives of their family type.

## 5. Conclusions

Overall, the findings of this study indicate that solo fathers and mothers accessed the same types of support, had similar reasons for becoming solo parents, and showed no differences in their mental health, or perceived social support. Concerning fathers’ experiences of social reactions to their family type, the findings showed that the fathers simultaneously experienced social approval and stigma. However, they showed resilience to the negative reactions of others.

## Figures and Tables

**Table 1:** Demographic characteristics.

	Solo Mothers (*n* = 19)		Solo Fathers (*n* = 17)		Independent Samples *t*-Test		
	*M*	*SD*	*M*	*SD*	*t*		*p*
Parent Age	44.05	3.66	47.18	5.81	1.90		*ns*
Child Age (months)	50.37	2.34	36.12	19.97	−2.93		>0.01
	*n*	*%*	*n*	*%*	Chi-square		
Child gender					*χ^2^*	*df*	*p*
Girl	10	(53%)	6	(35%)	1.09	1	*ns*
Boy	9	(47%)	11	(65%)			
Number of children							
One	10	(53%)	10	(59%)	1.90	2	*ns*
Two	7	(37%)	7	(41%)			
Three	2	(10%)	0				
Work status							
Not in employment	5	(26%)	3	(18%)	0.98	2	*ns*
Part-time	7	(37%)	5	(29%)			
Full time	7	(37%)	9	(53%)			

**Table 2:** Correlations between variables.

Variable	1.	2.	3.	4.	5.	6.
1. Parent Age	-					
2. Child Age	0.04	-				
3. Social Support	−0.06	0.09	-			
4. EDS	−0.03	−0.06	−0.33	-		
5. TAI	−0.02	−0.01	−0.38 *	0.71 **	-	
6. PSI	−0.09	−0.08	−0.31	0.33 *	0.38 *	-

* *p* < 0.05, ** *p* < 0.001.

**Table 3:** Reasons for becoming a solo parent.

Reason	Mothers (*n* = 19)	Fathers (*n* = 17)	Example Quotes
I wanted to be a mother/father	8 (42%)	6 (35%)	Mother ‘it’s just one of those natural things in life, becomes … you get to the point where that becomes something important to you.’ Father ‘It’s the joy of having babies and children, it brings so much happiness to a home and…and being someone for someone that needs your help and watching this little baby and then child through life.’
Timing felt right	2 (11%)	4 (23%)	Mother ‘I was just like right, I’ll fit this into my life now, and two sort of failed relationships, I was like, this is not gonna work!” Father ‘What I really wanted is to build a family but I will not wait or bring this to a next relationship as a must-have and a burden so I decided that maybe it’s time I do it on my own.’
I was getting older/time was running out	4 (21%)	2 (12%)	Mother ‘It always seemed like a um, I’m too young and it’s, you know, something I’ll do later, um, until you realise that well actually I ought to do something about it now.’ Mother ‘Just because of the clock ticking I spose, yeah’ Father ‘Age. Just doing the maths.’ Father ‘Well it was now or never really.’
I was financially secure	0	3 (18%)	Father ‘I was just ready. Like really ready, like financially stable, you know…got properties, I could afford it, because it isn’t cheap.’
All my friends were having children	1 (5%)	0	Mother ‘Cos friends were having them as well.’
Other	4 (21%)	2 (12%)	Mother ‘I didn’t like to see my son on his own, cos he was playing alone and I didn’t like to see it. It broke my heart watching him alone.’ Father ‘ [Described important family event]… I sort of suddenly desperately wanted to have children, and I was really surprised myself.’

**Table 4:** Discussions with others about becoming a solo parent.

	Mothers (*n* = 19)		Fathers (*n* = 17)		Example Quotes
	Yes	No	Yes	No	
Discussed with family	11 (58%)	8 (42%)	12 (71%)	5 (29%)	Mother ‘I spoke to everybody before I went through that loop. Oh god yeah, I spoke to my mum and my dad…then I spoke to my brother.’ Father ‘It was my family, I went to my family when I had already done the research, so I had chosen the agency and all of that, so my family was very supportive.’
Discussed with friends	14 (74%)	5 (26%)	14 (82%)	3 (18%)	Mother ‘Friends, yeah, not with my dad.’ Father ‘All my friends over and over and over and over, yes.’
Discussed with others (including support groups)	8 (42%)	11 (58%)	9 (53%)	8 (47%)	Mother ‘Didn’t speak to my parents, saw a counsellor who was really helpful.’ Father ‘Yeah, Facebook groups, single gay dads, single dads.’

**Table 5:** Means, SDs, *t* and *p* values for comparisons of mothers’ and fathers’ scores on the Multidimensional Scale of Perceived Social Support.

	Solo Mothers (*n* = 19)		Solo Fathers (*n* = 17)		*t*	*p*
	*M*	*SD*	*M*	*SD*		
MSPSS	5.08	1.11	5.30	1.37	0.55	*ns*

**Table 6:** Means, SDs, *t* and *p* values comparisons between mothers’ and fathers’ scores on the Edinburgh Depression Scale, the Trait Anxiety Scale and the Parenting Stress Inventory.

	Solo Mothers (*n* = 19)		Solo Fathers (*n* = 17)		*t*	*p*
	*M*	*SD*	*M*	*SD*		
EDS	4.89	3.45	3.71	2.42	−1.56	*ns*
TAI	34.89	8.47	32.12	5.69	129(U)	*ns*
PSI	60.28	13.73	57.29	8.54	−7.42	*ns*

## Data Availability

Data are contained within the article. The whole dataset is not publicly available due to ethical considerations.
